# Associations among NMR-measured inflammatory and metabolic biomarkers and accelerated aging in cardiac catheterization patients

**DOI:** 10.18632/aging.205758

**Published:** 2024-04-23

**Authors:** Henry Raab, Elizabeth R. Hauser, Lydia Coulter Kwee, Svati H. Shah, William E. Kraus, Cavin K. Ward-Caviness

**Affiliations:** 1 Center for Public Health and Environmental Assessment, US Environmental Protection Agency, Chapel Hill, NC 27514, USA; 2 Duke University Molecular Physiology Institute, Duke University, Durham, NC 27701, USA

**Keywords:** biological aging, NMR, biomarkers, cardiac catheterization

## Abstract

Research into aging has grown substantially with the creation of molecular biomarkers of biological age that can be used to determine age acceleration. Concurrently, nuclear magnetic resonance (NMR) assessment of biomarkers of inflammation and metabolism provides researchers with new ways to examine intermediate risk factors for chronic disease. We used data from a cardiac catheterization cohort to examine associations between biomarkers of cardiometabolic health and accelerated aging assessed using both gene expression (Transcriptomic Age) and DNA methylation (Hannum Age, GrimAge, Horvath Age, and Phenotypic Age). Linear regression models were used to associate accelerated aging with each outcome (cardiometabolic health biomarkers) while adjusting for chronological age, sex, race, and neighborhood socioeconomic status. Our study shows a robust association between GlycA and GrimAge (5.71, 95% CI = 4.36, 7.05, *P* = 7.94 × 10^−16^), Hannum Age (1.81, 95% CI = 0.65, 2.98, *P* = 2.30 × 10^−3^), and Phenotypic Age (2.88, 95% CI = 1.91, 3.87, *P* = 1.21 × 10^−8^). We also saw inverse associations between apolipoprotein A-1 and aging biomarkers. These associations provide insight into the relationship between aging and cardiometabolic health that may be informative for vulnerable populations.

## INTRODUCTION

The U.S. Census Bureau projects that by 2060, the life expectancy of Americans will increase by about six years to 85.6 and by 2034, individuals >65 years will outnumber children under 18 for the first time [1]. As humans age, the risk for disease increases [2], which makes understanding links between aging and early biomarkers of disease onset important [3]. However, the aging process is complex, and chronological age is an incomplete surrogate for biological aging [4]. Recently developed DNA methylation (DNAm) age biomarkers are a better predictor of biological age than chronological age [5], and DNAm age acceleration, the difference between DNAm age and chronological age, has been associated with all-cause mortality, cancer, severe sleep-disordered breathing, cognitive decline, cardiovascular disease, and several other health outcomes [6–10]. Cardiovascular disease (CVD), one of the leading causes of death worldwide [11, 12], inflammation, and metabolic dysfunction [13] are linked to aging [14–16].

Nuclear magnetic resonance (NMR) provides a novel means by which to detect metabolic and inflammatory biomarkers and other cardiometabolic disease risk factors [17, 18]. NMR spectra of human serum can be used to predict both the occurrence and severity of diseases like coronary heart disease (CHD) [19], and detect novel glycoprotein biomarkers of disease risk and inflammation, such as GlycA [20–22].

There are several molecular biomarkers of biological age. The properties of each biomarker, also called a “clock,” depend largely on how they are developed. Horvath’s pan-tissue epigenetic clock was developed using DNA methylation measured on multiple tissues and cell types [23]. The Horvath pan-tissue clock accurately estimates epigenetic age of several tissues and is strongly associated with mortality and disease risk [4]. Blood-specific DNA methylation based aging biomarkers include Hannum Age [24], GrimAge [25], and Phenotypic Age [26]. These measures were all developed differently and are described in more detail in the Methods section. Despite differences in their construction all of these aging biomarkers have been strongly associated with morbidity and mortality though the magnitude of these associations can differ.

In addition to their method of development, the underlying ‘omics data used can have a substantial impact on the properties of a given aging clock. Horvath, Hannum, GrimAge, and Phenotypic Age were all developed using DNA methylation – the primary molecular data type for aging clocks since the publication of the Horvath pan-tissue clock [23]. Gene expression (the cellular transcriptome) is also used to create robust and highly predictive aging biomarkers. Transcriptomic Age, is an aging biomarker based on gene expression data [27] and accelerated Transcriptomic Age is associated with blood pressure, cholesterol levels, fasting plasma glucose, and body mass index [27].

Though there are robust associations between chronological age and measures related to inflammation and metabolic dysfunction, associations between these measures and epigenetic aging biomarkers show some heterogeneity. The GOLDN study found associations between HDL and accelerated epigenetic aging (Horvath and Hannum clocks) but not LDL or total cholesterol [28]. Most studies of epigenetic aging and metabolic or inflammatory biomarkers are done in young, healthy adults and show that epigenetic age acceleration is associated with triglyceride and HDL concentrations, as well as inflammatory and metabolic markers, like C-reactive protein [29, 30]. However, there is limited information on associations between metabolic outcomes and epigenetic aging across the life course or among those with pre-existing disease. In a recent study of cardiac catheterization patients, associations between accelerated epigenetic aging and mortality were partially mediated by diabetes, indicating that links between aging and metabolic dysfunction are vital relationships to understand in this population [31]. This study utilizes the CATHGEN cohort from the Jiang et al. study to investigate associations between multiple epigenetic and transcriptomic aging biomarkers and a broad array of NMR-based measures of inflammation, lipid homeostasis, and diabetes risk.

## RESULTS

### Cohort description

**Figure 1:**
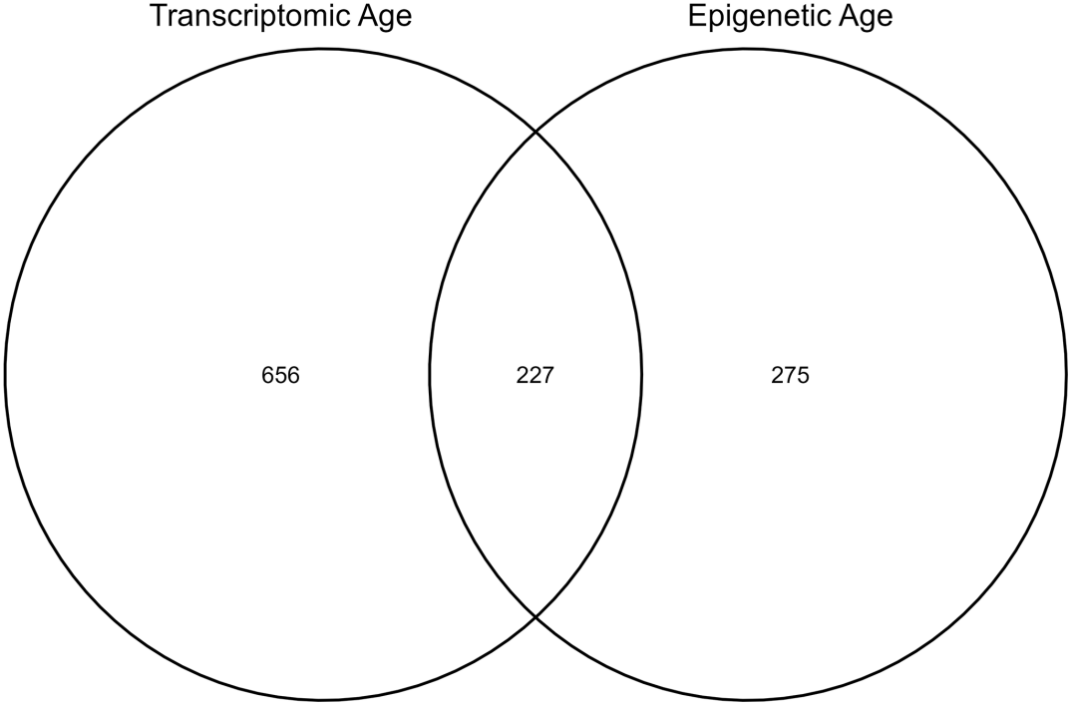
Number of individuals in the sub-cohorts for each analysis.

Within our study cohort, there were 1284 CATHGEN participants with gene expression data, of which 883 also had NMR data. There were also 563 CATHGEN patients with DNA methylation data, of which 502 had NMR data. Of those CATHGEN participants with DNA methylation data, 227 overlapped with the 883 individuals with gene expression data. To evaluate associations between epigenetic age and NMR measures we used all 502 participants with both sets of data, while for analyses of transcriptomic age and NMR measures we used all participants with transcriptomic and NMR data available (*N* = 883). In joint analyses of both transcriptomic and epigenetic age, we were restricted to the 227 overlapping samples (Figure 1). Participants in the three sub-cohorts were similarly aged at just over 60 years old, with slightly higher ages in the group with combined transcriptomic and DNA methylation data (Table 1). The transcriptomic aging group was less urban, had a higher percentage of white participants, and was slightly less educated than the other two groups. Participants with DNA methylation data available, including those with both DNA methylation and gene expression, had higher home values, household income, and a lower percentage of smokers than all study participants with gene expression data.

**Table 1:** Description of the study cohort.

	**Gene expression (*n* = 883)**	**DNAm (*n* = 502)**	**Gene expression and DNAm (*n* = 227)**
	**Mean (± SD)**	**Mean (± SD)**	**Mean (± SD)**
Chronological Age (y)	62.2 ± 11.9	60.5 ± 12.5	63.2 ± 12.4
Accel Grim Age (y)		−2.2 ± 6.4	−2.4 ± 6.6
Accel Hannum Age (y)		11.2 ± 7.0	9.8 ± 6.9
Accel Horvath Age (y)		4.7 ± 6.7	3.8 ± 6.6
Accel Phenotypic Age (y)		−8.9 ± 7.6	−9.6 ± 7.3
Accel Transcriptomic Age (y)	−6.5 ± 11.5		−8.1 ± 12.0
Poverty (%)	19.7 ± 14.9	21.4 ± 18.5	18.4 ± 18.0
Urbanicity (%)	55.4 ± 44.2	83.3 ± 33.5	85.9 ± 30.2
HS Education or more (%)	82.5 ± 11.5	84.9 ± 12.8	86.8 ± 12.5
Median household value ($)	150 676 ± 93 805	200 145 ± 102 107	210 200 ± 104 069
Median household income ($)	48 367 ± 24 553	53 927 ± 29 848	58 726 ± 30 292
	*N* (%)	*N* (%)	*N* (%)
Males (N)	346 (39.2)	284 (56.5)	101 (44.5)
Females (N)	537 (60.8)	218 (43.5)	126 (55.5)
White (N)	628 (71.1)	315 (62.7)	150 (66.1)
Black (N)	198 (22.4)	187 (37.3)	77 (33.9)
Other (N)	57 (6.5)	0 (0)	0
History of Smoking (Yes) (N)	429 (48.5)	221 (44.0)	101 (44.5)

Accel ages (accelerated ages) refer to the difference between calculated age and chronological age. Data are reported as mean ± SD or number (n) and the percentage (%). Data is reported in years (y), percent (%), dollars ($), or counts (N). Abbreviation: DNAm: DNA methylation.

### Transcriptomic age estimation

**Figure 2:**
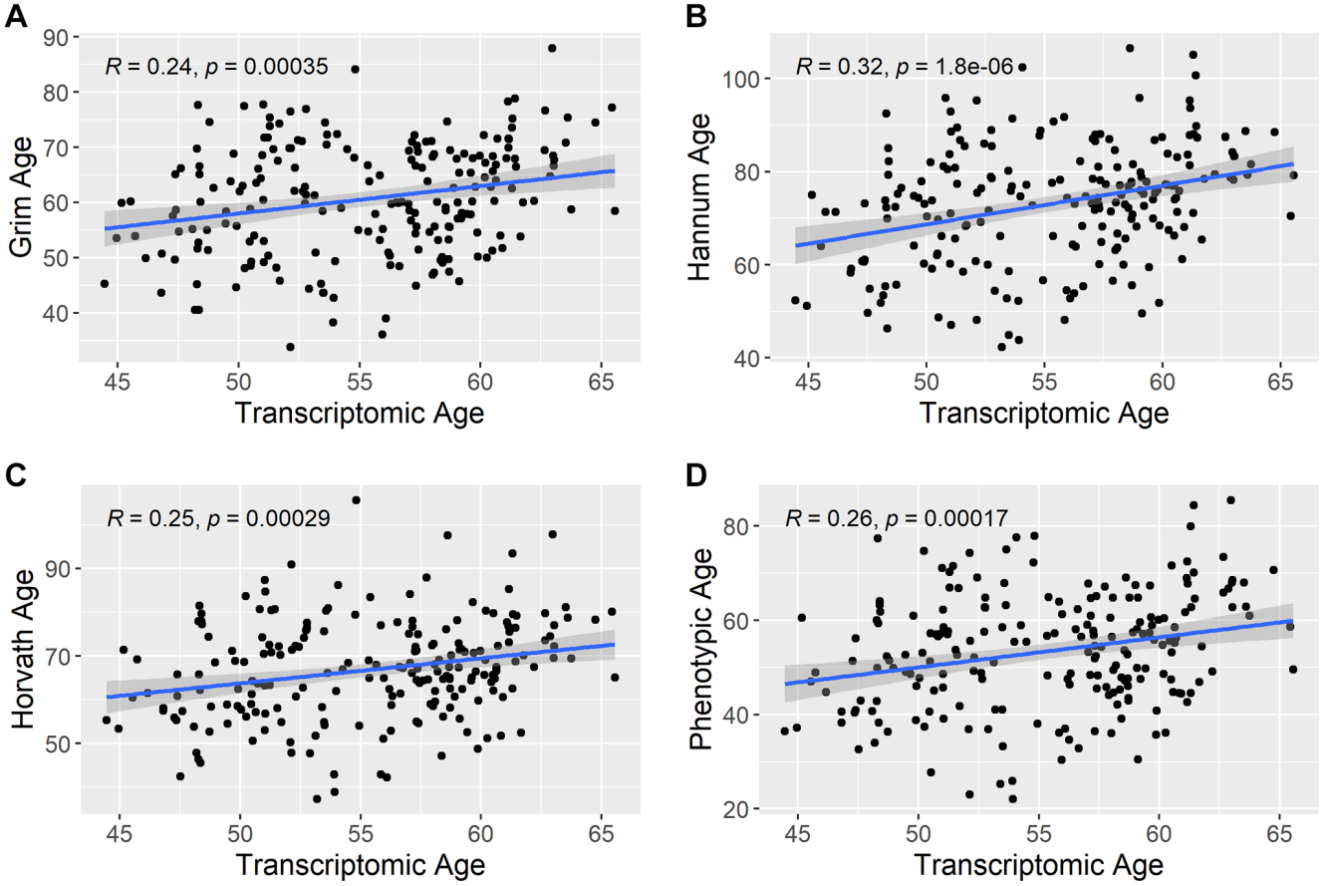
**Correlation between transcriptomic age and other epigenetic aging predictors.** The scatterplots show the correlation between estimated transcriptomic age and the other epigenetic aging predictors in the study. (**A**) shows the correlation between Transcriptomic Age and Grim Age, (**B**) shows the correlation between Transcriptomic Age and Hannum Age, (**C**) shows the correlation between Transcriptomic Age and Horvath Age, and (**D**) shows the correlation between Transcriptomic Age and Phenotypic Age. R = Pearson correlation coefficient.

There are multiple ways to estimate Transcriptomic Age based on the type of confounder adjustment done during its estimation. Three approaches to estimate Transcriptomic Age were explored here: No confounder adjustment (intercept only model), sex adjusted, sex and smoking status adjusted, and sex, smoking status, and cell count adjusted. This was done to explore the degree to which various confounder adjustments impacted the correlation of Transcriptomic Age with chronological age. There was no difference in the correlation with chronological age for Transcriptomic Age estimated using the intercept only model versus Transcriptomic Age estimated while adjusting for sex only (Pearson r = 0.29). This was also the same correlation when estimating Transcriptomic Age using a sex and smoking-status-adjusted model. Correlations between all transcriptomic age estimation methods and chronological age are given in Supplementary Figure 1. The correlation between Transcriptomic Age and chronological age decreased when the model was adjusted for cell counts (Pearson r = 0.13). Therefore, we used Transcriptomic Age estimated based on residuals from regressing sex and smoking status regressed on the gene expression probes for all analyses. The correlation between Transcriptomic Age and other epigenetic aging predictors is shown in Figure 2. All epigenetic aging biomarkers (GrimAge, Hannum, Horvath, and Phenotypic age) were estimated using the online calculator at https://dnamage.genetics.ucla.edu/home. GrimAge, Hannum Age, Horvath Age, and Phenotypic Age were correlated with Transcriptomic Age with Pearson r = 0.24, 0.32, 0.25, 0.26, respectively.

### Associations between NMR biomarkers and aging measures

**Figure 3:**
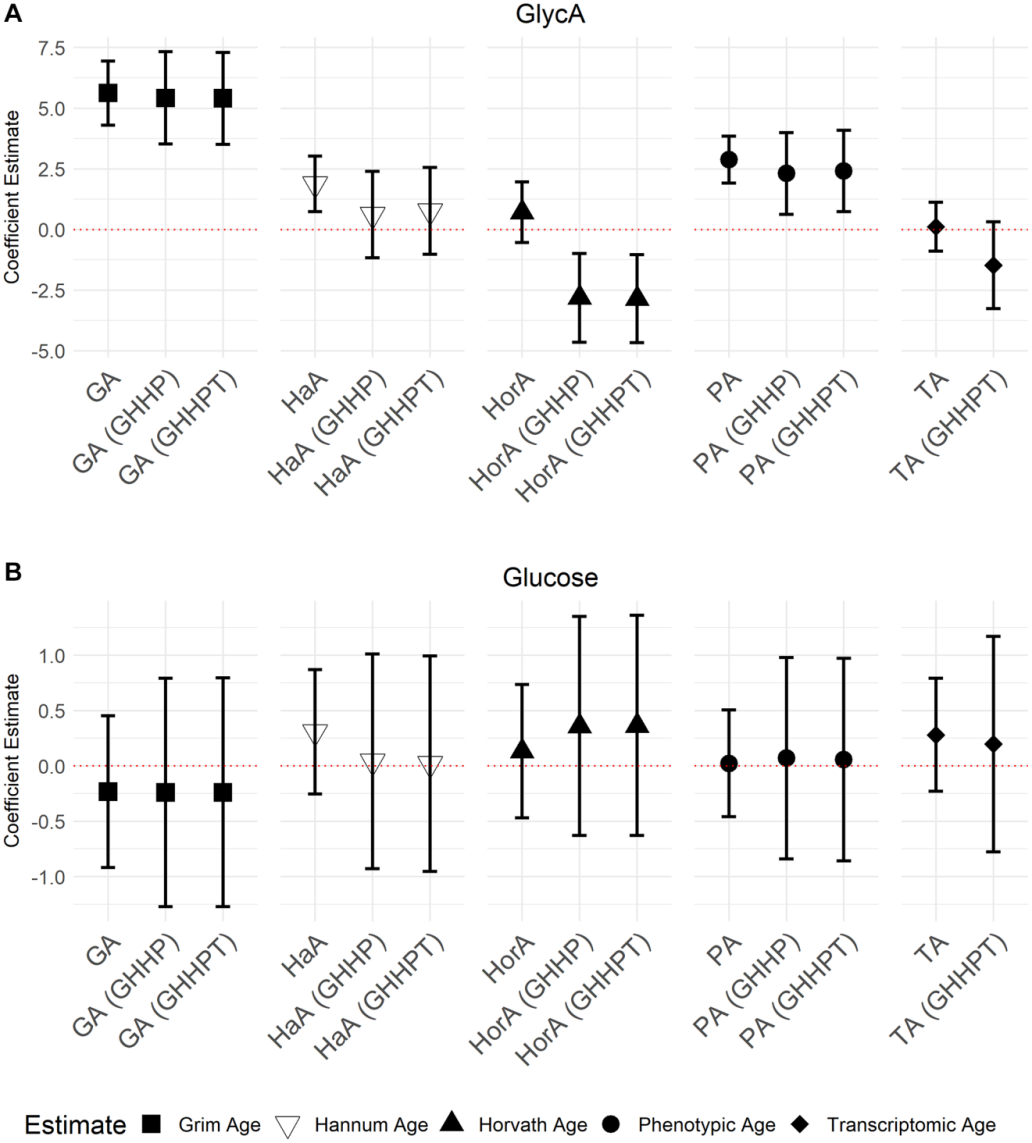
**Models of associations between GlycA and glucose NMR biomarkers and accelerated ages of epigenetic and transcriptomic biomarkers.** Models were run with aging biomarkers (Abbreviations: GA: GrimAge; HaA: Hannum Age; HorA: Horvath Age; PA: Phenotypic Age; TA: Transcriptomic Age) and then adjusted for other aging biomarkers. Models with epigenetic aging biomarkers were adjusted for GrimAge, Hannum Age, Horvath Age, and Phenotypic Age (GHHP). Models with transcriptomic and epigenetic biomarkers were adjusted for GrimAge, Hannum Age, Horvath Age, Phenotypic Age, and Transcriptomic Age (GHHPT). Error bars represent a 95% confidence interval. (**A**) is the association between GlycA and the aging predictors and (**B**) displays the association between glucose and the aging predictors.

**Figure 4:**
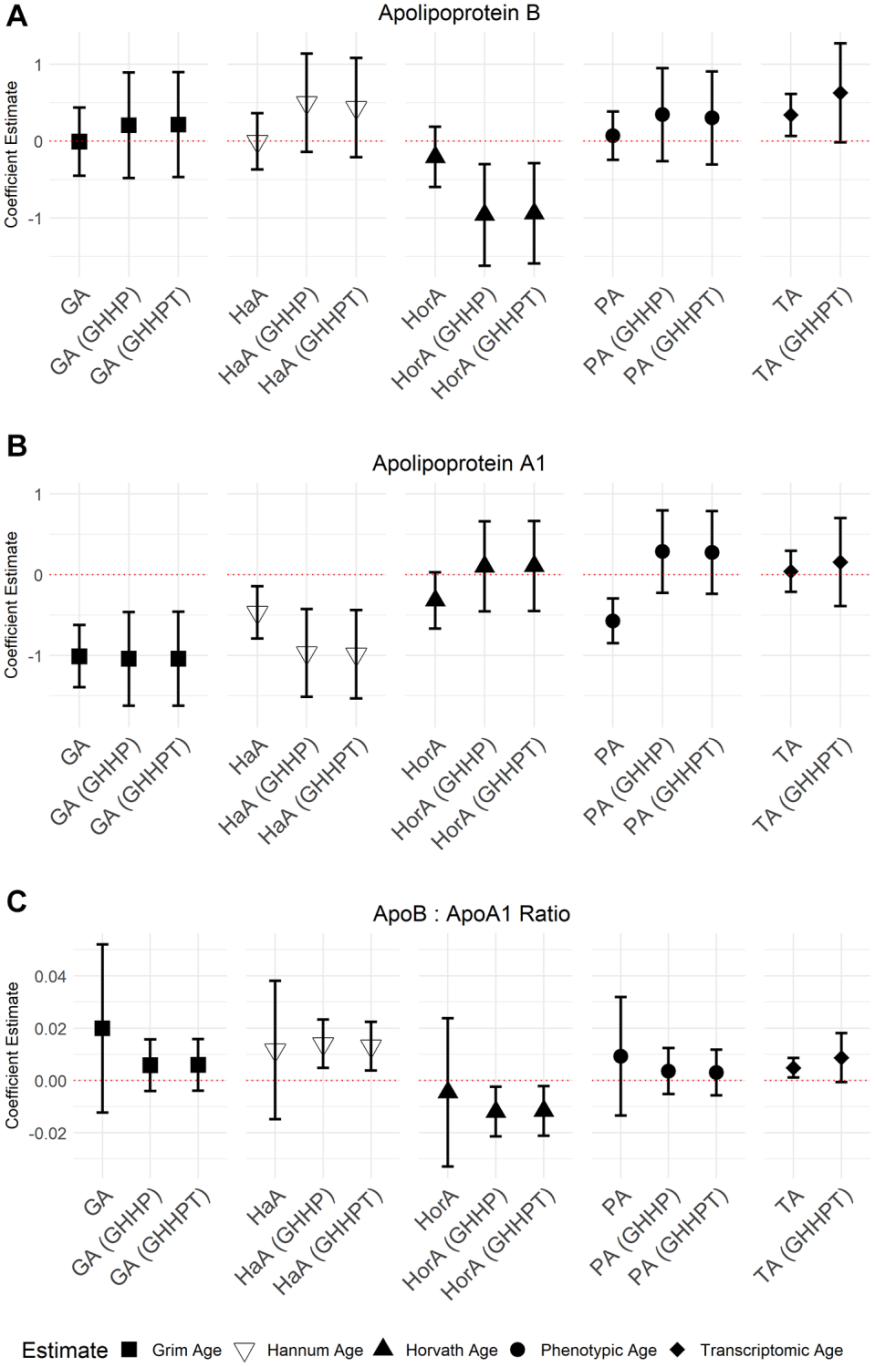
**Models of associations between ApoB, ApoA-1, and the ApoB: A-1 ratio NMR biomarkers and accelerated ages of epigenetic and transcriptomic biomarkers.** Models were run with aging predictors (Abbreviations: GA: GrimAge; HaA: Hannum Age; HorA: Horvath Age; PA: Phenotypic Age; TA: Transcriptomic Age) and then adjusted for other aging biomarkers. Models with accelerated ages of epigenetic aging biomarkers were adjusted for GrimAge, Hannum Age, Horvath Age, and Phenotypic Age (GHHP). Models with accelerated ages of transcriptomic and epigenetic biomarkers were adjusted for GrimAge, Hannum Age, Horvath Age, Phenotypic Age, and Transcriptomic Age (GHHPT). Error bars represent a 95% confidence interval. (**A**–**C**) show the association between Apolipoprotein B, Apolipoprotein A-1, and the ApolipoproteinB: A-1 ratio and the aging predictors, respectively.

GrimAge (5.71; 95% CI = 4.36, 7.05, *P* = 7.94 × 10^−16^), Hannum Age (1.81; 95% CI = 0.65, 2.98, *P* = 2.30 × 10^−3^), and Phenotypic Age (2.88; 95% CI = 1.91, 3.87, *P* = 1.21 × 10^−8^) acceleration were all positively associated with GlycA (Figure 3). We also observed a negative association between apoA-1 and GrimAge (−1.03; 95% CI = −1.42, −0.63, *P* = 5.50 × 10^−7^), Hannum Age (−0.41; 95% CI = −0.74, −0.08, *P* = 0.01), and Phenotypic Age (−0.55; 95% CI = −0.83, −0.26, *P* = 1.58 × 10^−4^) acceleration (Figure 4). In contrast, Transcriptomic Age acceleration was positively associated with apoA-1 (0.34, 95% CI = 0.04, 0.63, *P* = 0.03). No aging predictors were associated with blood glucose concentrations. We examined the VIF for all models and saw a slight elevation of VIF in models with accelerated Transcriptomic Age but not others using the common VIF >5 cutoff. All results are in Supplementary Table 1.

In models adjusted for all epigenetic and transcriptomic aging predictors, accelerated GrimAge remained positively associated with GlycA (5.03, 95% CI = 3.01, 7.05, *P* = 1.85 × 10^−6^) and inversely associated with apoA-1 (−1.13, 95% CI = −1.71, −0.55, *P* = 1.83 × 10^−4^). These associations were the only Bonferroni significant (*P* < 0.05/35) associations seen in the models with multiple aging predictors. There were several associations with *P* < 0.05 including between GrimAge and apoB: A-1 ratio (0.01, 95% CI = −1.16, 0.99, *P* = 6.84 × 10^−3^) (Figures 3 and 4; Supplementary Table 2). Inverse associations between accelerated Hannum Age and apoA-1 also remained, while Phenotypic Age maintained its positive association with GlycA. The only association observed in the models adjusted for all epigenetic and transcriptomic aging biomarkers that were not observed in the individual models was a negative association between accelerated Horvath Age and GlycA (−2.94, 95% CI = −4.86, −1.02, *P* = 2.89 × 10^−3^) and Horvath Age and ApoB (−0.80, 95% CI = −1.43, −0.16, *P* = 0.01). Results for these models are in Supplementary Table 2.

Given that only a subset of the participants had both epigenetic and transcriptomic data (Table 1) we also ran models with all epigenetic aging biomarkers but without Transcriptomic Age included. Results were similar to those observed when Transcriptomic Age was also included indicating that the addition of transcriptomic age, and subsequent sample size restriction, did not substantially impact associations (Supplementary Table 3). After adjustment for the epigenetic aging biomarkers, accelerated Transcriptomic Age was not associated with any of the NMR biomarkers and had an elevated VIF (>5) in all models suggesting that the results for this model may be inflated, likely due to high correlations amongst the predictors and the relatively small sample size.

### Associations between LP-IR, DRI and aging measures

**Figure 5:**
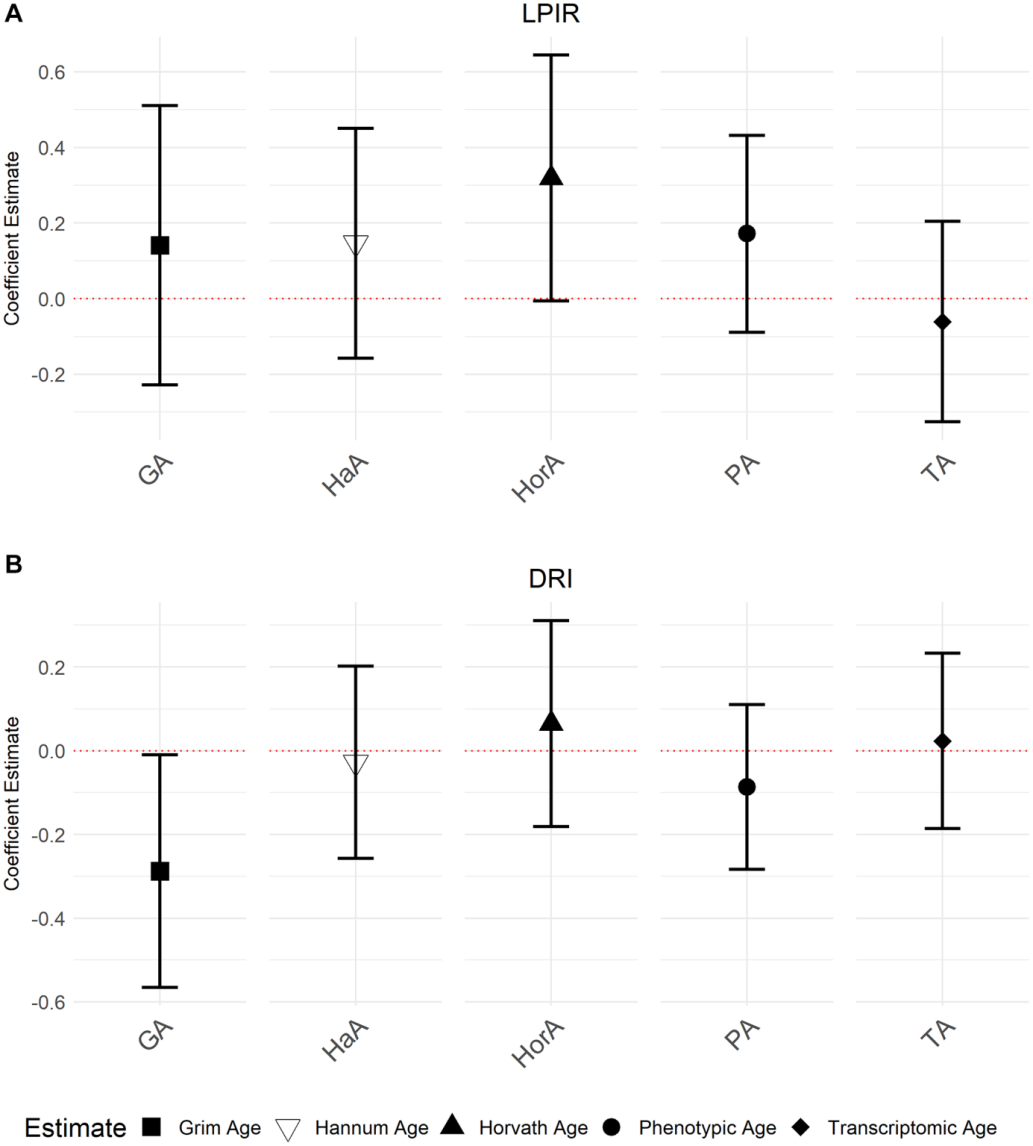
**Associations between NMR biomarkers LP-IR and DRI and the accelerated ages of epigenetic and transcriptomic biomarkers.** Models were run with accelerated ages of epigenetic and transcriptomic biomarkers (Abbreviations: GA: GrimAge; HaA: Hannum Age; HorA: Horvath Age; PA: Phenotypic Age; TA: Transcriptomic Age). (**A**) is the association between LP-IR and the aging predictors and (**B**) displays the association between DRI and the aging predictors. Error bars represent 95% confidence intervals.

We also investigated associations between aging biomarkers and the NMR biomarkers LP-IR (Lipoprotein Insulin Resistance Index) and DRI (Diabetes Risk Index) which combine multiple NMR measures to inform on metabolic risks. Accelerated GrimAge was inversely associated with DRI (−0.29, 95% CI = −0.57, −2.03 × 10^−3^, *P* = 0.05). Although not statistically significant, there was weak evidence for a positive association between accelerated Horvath Age and LP-IR (0.32, 95% CI = −9.36 × 10^−3^, 0.66; *P* = 0.06). We did not observe any other associations (Figure 5; Supplementary Table 4). We also did not observe independent associations when including all aging biomarkers in the same model (Supplementary Table 5), including when excluding Transcriptomic Age from the models as done before (Supplementary Table 6). As before, there was evidence for multi-collinearity for accelerated Transcriptomic Age in the model, including Transcriptomic Age and all the epigenetic aging predictors (Supplementary Table 5), warranting substantial caution in interpreting these results.

### Associations between LDL, HDL and aging measures

Finally, we also examined NMR measures of LDL and HDL as secondary outcomes, as these outcomes have not been examined for individuals with underlying cardiovascular disease [32–34]. Hannum Age (−0.30, 95% CI = −0.65, 6.28 × 10^−3^, *P* = 0.05) and accelerated Phenotypic Age (−0.26, 95% CI = −0.56, −0.04, *P* = 0.03) was negatively associated with HDL. Both Hannum Age (−1.11, 95% CI = −2.16, −0.07, *P* = 0.04) and Horvath Age (−1.13, 95% CI = −2.15, −0.10, *P* = 0.03) were negatively associated with LDL (Supplementary Table 7).

## DISCUSSION

Cardiovascular and metabolic diseases are some of the most common age-related diseases and are projected to grow in prevalence given the aging of the United States population [15, 35, 36]. While advanced chronological age is a risk factor CVD and metabolic dysfunction, biological aging biomarkers are better tied to intrinsic biological processes and are associated with morbidity and mortality independent of chronological age [37–40]. Novel NMR-based biomarkers may be used to identify risk factors for disease; however, there are still significant gaps in the research on the relationship between these novel NMR biomarkers and aging biomarkers [22, 40–42]. We address this data gap in this study by reporting associations with epigenetic and transcriptomic age for many of these biomarkers and utilizing a study cohort of cardiac catheterization patients with elevated morbidity and mortality risks. We decided to use GrimAge, Horvath Age, Hannum Age, and Phenotypic Age as these clocks represent both first generation (Hannum and Horvath Age) and second generation (GrimAge, Phenotypic Age) clocks allowing us to evaluate how clocks derived with different approaches associate with these biomarkers. As this was not meant to be an exhaustive study of all epigenetic clocks, we did not include every clock published in the literature. While assessing biological age using DNA methylation is one of the most common methods, aging is a complex process that that is likely to be reflected in multiple molecular hierarchies, e.g., DNA methylation, gene transcription, and even protein concentrations. For this reason we also evaluated associations with transcriptomic age which has been shown to have different associations with clinical outcomes than DNA methylation age, and thus may also show associations with biomarkers of inflammation and metabolism that differ from DNA methylation-based aging biomarkers in informative ways.

We observed an association between GrimAge, an estimator of lifespan and all-cause mortality [25, 43], and GlycA, a biomarker for systemic inflammation and CVD risk [22]. Studies show that GlycA is also positively associated with C-reactive protein (CRP) [44], a traditional biomarker for inflammation [45]. GlycA and high-sensitivity CRP (hs-CRP) have a similar predictive value for CVD-related events [21]. While both GlycA and hs-CRP are used to measure inflammation, GlycA is a composite biomarker that may have some advantages over a single molecule like hs-CRP, like being a more stable measurement and low intra-individual variability [46]. However, more research is needed to explore the utility of GlycA as compared to traditional inflammation biomarkers such as hs-CRP. We observed an inverse relationship between GrimAge and DRI suggesting that increasing GrimAge is associated with a lower diabetes risk according to the DRI. It’s worth nothing that this association was in the opposite direction of other associations with GrimAge, e.g., GlycA, but nonetheless could indicate a more complex relationship between GrimAge and DRI – possibly driven by diabetes treatment status or mediation which we could not account for in this study. Further investigations of GrimAge and diabetes risk are warranted to better understand this relationship.

ApoA-1 and apoB carry lipids and cholesterol in the blood [47]. We did not observe an association with apoB, but apoA-1 was inversely associated with accelerated GrimAge, Hannum Age, and Phenotypic Age. Given that apoA-1 is cardioprotective [48, 49] an inverse association between apoA-1 and epigenetic aging biomarkers is not unexpected as there is a positive association between accelerated epigenetic age and CVD. Research has shown that plasma apoA-1 increases with age [50] and is an indicator of cardiovascular risk [51].

Previous studies have shown that commonly used metabolic biomarkers, like HDL, are associated with epigenetic age acceleration. In the GOLDN study, HDL was inversely associated with Horvath and Hannum age acceleration [28]. Meanwhile, in a coronary artery risk development study (CARDIA) HDL was inversely associated with GrimAge acceleration [29]. We see a similar pattern in the secondary analysis of HDL in our study which shows inverse associations between HDL and Hannum and Phenotypic age acceleration as well as weaker inverse associations with Horvath age and GrimAge acceleration. The GOLDN study also shows a pattern for stronger positive associations with inflammatory biomarkers especially with Hannum age acceleration. The same pattern is present in our study with positive associations between GlycA and GrimAge, Hannum age, and Phenotypic age acceleration, and to a lesser extent, Horvath age acceleration. This suggests that our results are not a cohort specific effect and may not be limited to those with underlying cardiovascular disease.

There are several strengths and limitations of this study. Although we did have a moderately large sample size for the patients with gene expression data (*N* = 883), the number of patients with DNAm data (*N* = 502) and matching transcriptomic and DNAm data (*N* = 227) were smaller. However, the consistency of the results between the epigenetic and epigenetic + transcriptomic datasets shows that the smaller sample size is not much of an issue. We did not have information on medications which could be an important confounder that modifies some of the biomarkers examined. The relationship between medication usage and accelerated aging is also unknown so future studies that are able to evaluate the role of medications in these associations will substantially add to our understanding of aging biomarkers. In addition to medications, further information on behavioral factors, such as alcohol usage – which was not available in CATHGEN, could help to refine associations. Considering the study was limited to patients that had undergone cardiac catheterization, associations between NMR lipoprotein biomarkers and epigenetic and transcriptomic age acceleration may not generalize to other populations. However, previous studies with other cohorts show a similar pattern of results with weaker associations between metabolic biomarkers and aging biomarkers and stronger associations between inflammatory biomarkers and aging biomarkers [28, 29]. This study is the first to examine associations between these novel NMR lipoprotein biomarkers and multiple epigenetic and transcriptomic aging predictors, especially in a population enriched for individuals with CVD. A limitation of this study is the lack of medication information available. We observed no associations between glucose and aging measures and inverse associations between GrimAge and DRI. It is possible that medication usage partially explains these observations and is a factor that should be incorporated into future studies of aging and metabolic traits.

In conclusion, this study is an initial examination of the associations between epigenetic and transcriptomic aging biomarkers and novel NMR lipoprotein biomarkers. As researchers work to better understand the clinical significance of aging biomarkers, work to understand their relationship with known markers of inflammation and metabolism can better establish their utility in clinical medicine. Additionally, as these NMR biomarkers continue to be evaluated as subclinical risk factors, it is crucial to understand their associations with biological aging measures which may aid in identifying aging-related disease biomarkers. Taken together these insights will aid in understanding the links between biological aging and health, especially among clinically vulnerable populations.

## METHODS

### Study population

We used data collected from cardiac catheterization patients at Duke University Hospital in North Carolina between 2001–2010 (CATHGEN, CATHeterization GENetics Study) [52]. Peripheral blood was collected during the cardiac catheterization procedure per previously published methods. Enrollment in CATHGEN included linkage with existing patient medical records allowing for the extraction of demographics, pre-existing co-morbidities, clinical labs, and vital measurements.

### Transcriptomic age calculation

Whole genome gene expression data was captured for 1284 CATHGEN individuals [52]. Assessment of quantitative whole genome RNA data was done using the Human HT-12v3 Expression BeadChip (Illumina, San Diego, CA, USA), and quality control (QC) was done using Illumina GenomeStudio [52]. Probes detected in more than 50% of samples with a detection *P*-value of < 0.05 were added to the CATHGEN database for 1284 samples. In these samples, 12,800 probes passed detection and QC filters.

Transcriptomic Age acceleration measures the difference between estimated and chronological age with a net positive meaning age acceleration. Estimating Transcriptomic Age is a two-step process. First, residuals were estimated for each gene expression probe. The original Transcriptomic Age predictor by Peters et al. used a ridge regression penalized model on the entire gene expression array (Illumina Infinium HumanMethylation450k BeadChip Array). Of the 11,779 genes used in the original estimation model, 7,497 passed QC in CATHGEN. The weights from the Peters et al. (2015). Transcriptomic Age estimator was applied to the CATHGEN data in the second step. Non-overlapping probes were set to 0, as was done for validation cohorts in the original manuscript when the probes passing QC did not perfectly encompass that of the development cohort. The original predictor used a series of regression models to compare the behavior of the predictor ranging from using an intercept-only model to calculate the residuals up to a model accounting for age, sex, smoking status, and cell counts. We estimated Transcriptomic Age using an intercept-only regression model and ones adjusting for sex, sex + smoking status, and sex + cell counts + smoking status. We observed similar correlations between these models and chronological age, indicating the robustness of the residual estimation method (Supplementary Figure 1). The estimated cell counts used in the cell count adjustment model were monocytes, granulocytes, cytotoxic T-cells (CD8T), CD4 T lymphocytes, natural killer cells (NK), and B lymphocytes which were estimated using the Houseman method as implemented in the online epigenetic aging clock estimator (https://dnamage.genetics.ucla.edu/home) [53].

### Epigenetic age estimation

We used several different epigenetic aging predictors in our models, Hannum Age, GrimAge, Horvath Age, and Phenotypic Age. Hannum Age is a single-tissue DNAm age estimator that uses the percent methylation of 71 CpG markers in blood cells [24] to estimate age. DNAm GrimAge, or GrimAge, is a predictor of lifespan that uses the percent methylation at several hundred CpG loci along with chronological age and sex, as well as a DNAm estimator of smoking pack-years to obtain an aging estimate [25]. GrimAge can predict lifespan, health span, age-related clinical phenotypes, and all-cause mortality [25, 43]. Horvath Age is a unique aging predictor because it is valid for multiple tissues and cell types which assesses DNAm data from 353 CpG loci using a penalized regression [23]. Phenotypic Age is an aging predictor that is a robust estimator for morbidity and mortality outcomes that uses DNA methylation at 513 CpG loci to estimate age [26]. The loci were selected from those that best predicted nine age-associated clinical parameters. DNA methylation for the CATHGEN study was processed using the *methylumi* R package with normalization performed using normal-exponential convolution using out-of-band probes (noob) followed by quantile normalization which was found to perform as good or better than other methods for this data. The aforementioned epigenetic ages were estimated for the CATHGEN cohort using an online calculator (http://dnamage.genetics.ucla.edu/). For this study, we estimated age acceleration as the difference between epigenetic and transcriptomic and chronological age. Chronological age was included in all regression models making this approach equivalent to using the residuals-based age acceleration definitions also seen in the literature.

### NMR biomarkers

Cardiometabolic biomarkers were measured in 8,738 CATHGEN individuals using NMR spectroscopy at LipoScience, Inc., (Raleigh, NC, USA) [54, 55] performed on fasting plasma samples collected at the time of the catheterization procedure, but prior to the administration of heparin or initiation of the procedure specific actions. These NMR-measured metabolites and biomarkers included in this analysis were: GlycA, Apolipoprotein B (apoB), Apolipoprotein A1 (apoA-1), and glucose. We focused on these biomarkers as they have yet to be evaluated for associations with accelerated aging despite their links with age-related cardiometabolic disease. GlycA is a novel biomarker of systemic inflammation, and evidence shows that it is elevated in acute and chronic inflammation [22]. Apolipoprotein B (apoB) is a lipoprotein that can get trapped within the arterial wall, accelerating the atherosclerotic process [42, 56]. It is associated with insulin resistance [57] and myocardial infarction [58]. Apolipoprotein A1 (apoA-1) is the primary component in HDL-C [59], and evidence suggests it is inversely associated with type 2 diabetes and blood pressure in patients with coronary artery disease [60, 61]. We also calculated the apoB: apoA-1 ratio which is associated with metabolic syndrome [62] and coronary heart disease in obese patients [63].

In addition to the four biomarkers described above, we examined two NMR biomarkers of multiple NMR-measured features strongly associated with metabolic dysfunction. LP-IR and DRI were both estimated using lipoprotein data measured via NMR [41, 64]. LP-IR is calculated by adding weighting scores of six different lipoprotein particles [54, 64], and has been associated with type 2 diabetes mellitus [65] and insulin resistance [64]. DRI was calculated with logistic regression to find the regression coefficients in a model with LP-IR and a branch-chain amino acid (BCAA) parameter (valine + 2 × leucine) [41]. Valine and leucine were used because of their associations with future diagnoses and risk of type-2 diabetes [66, 67].

### Statistical analysis

We used linear regression to investigate the associations between accelerated aging and NMR biomarkers. We used demographics from hospital records and socio-economic data from the 2010 US Census as confounders to adjust for age, sex, race, smoking status, and the following census block group variables: Median household income, median household value, poverty rate, urbanicity, and percentage of persons with a high school education or more. Smoking status was not included in the models with Transcriptomic Age due to its inclusion in the estimation of Transcriptomic Age. Confounders were chosen based on previous studies in CATHGEN [31, 68–70]. Although inflammatory and metabolic outcomes like body fat, C-reactive protein, and obesity have been associated with epigenetic aging these outcomes are directly related to our outcomes of interest and thus are within the causal pathway of interest. Thus, we did not adjust for metabolic or inflammatory outcomes as these would not be confounders and adjusting for them could bias associations.

The outcomes examined were GlycA, apoB, apoA-1, Glucose, and apoB: apoA-1 ratio, and the NMR multi-markers (LP-IR and DRI) with the aging measures as the independent variable of interest. As previous studies have not investigated these NMR biomarkers, we also examined HDL and LDL as secondary analyses included post-hoc during the literature review process. These outcomes were included to facilitate comparison with existing literature.

We ran models for each aging measure individually and then adjusted for multiple aging predictors to evaluate if aging predictors had independent associations. We examined one model that included all the epigenetic aging predictors and one that examined all the epigenetic aging predictors and transcriptomic age. Given the small overlap between the epigenetic and transcriptomic data, we examined both models to maximize sample size. We evaluated models with multiple aging predictors for multi-collinearity using the various inflation factor (VIF).

All models were run using R version 1.3.959, and the results are reported as the regression coefficient from the linear models along with the associated 95% confidence interval (CI). Given some analyses' relatively small sample sizes, we report all associations with *P* < 0.05. Bonferroni correction was applied to *P*-values to adjust for multiple testing. Associations that were Bonferroni significant after correcting for the five aging biomarkers and seven outcomes (*P* < 0.05/35 = 1.4 × 10^−3^) are noted in the Results and Discussion.

## Supplementary Materials

Supplementary Figure 1

Supplementary Tables

## References

[1] Medina L, Sabo S, Vespa J. Living Longer: Historical and Projected Life Expectancy in the United States, 1960 to 2060. Population Estimates and Projections: U.S. Census Bureau. 2020; 25–45.

[2] Harman D. The aging process: major risk factor for disease and death. Proc Natl Acad Sci U S A. 1991; 88:5360–3. 10.1073/pnas.88.12.5360 2052612 PMC51872

[3] Strimbu K, Tavel JA. What are biomarkers? Curr Opin HIV AIDS. 2010; 5:463–6. 10.1097/COH.0b013e32833ed177 20978388 PMC3078627

[4] Horvath S, Raj K. DNA methylation-based biomarkers and the epigenetic clock theory of ageing. Nat Rev Genet. 2018; 19:371–84. 10.1038/s41576-018-0004-3 29643443

[5] Salameh Y, Bejaoui Y, El Hajj N. DNA Methylation Biomarkers in Aging and Age-Related Diseases. Front Genet. 2020; 11:171. 10.3389/fgene.2020.00171 32211026 PMC7076122

[6] Perna L, Zhang Y, Mons U, Holleczek B, Saum KU, Brenner H. Epigenetic age acceleration predicts cancer, cardiovascular, and all-cause mortality in a German case cohort. Clin Epigenetics. 2016; 8:64. 10.1186/s13148-016-0228-z 27274774 PMC4891876

[7] Tan Q. Epigenetic age acceleration as an effective predictor of diseases and mortality in the elderly. EBioMedicine. 2021; 63:103174. 10.1016/j.ebiom.2020.103174 33340996 PMC7750548

[8] Li X, Joehanes R, Hoeschele I, Rich SS, Rotter JI, Levy D, Liu Y, Redline S, Sofer T. Association between sleep disordered breathing and epigenetic age acceleration: Evidence from the Multi-Ethnic Study of Atherosclerosis. EBioMedicine. 2019; 50:387–94. 10.1016/j.ebiom.2019.11.020 31761615 PMC6921369

[9] Vaccarino V, Huang M, Wang Z, Hui Q, Shah AJ, Goldberg J, Smith N, Kaseer B, Murrah N, Levantsevych OM, Shallenberger L, Driggers E, Bremner JD, Sun YV. Epigenetic Age Acceleration and Cognitive Decline: A Twin Study. J Gerontol A Biol Sci Med Sci. 2021; 76:1854–63. 10.1093/gerona/glab047 33606025 PMC8436988

[10] Roetker NS, Pankow JS, Bressler J, Morrison AC, Boerwinkle E. Prospective Study of Epigenetic Age Acceleration and Incidence of Cardiovascular Disease Outcomes in the ARIC Study (Atherosclerosis Risk in Communities). Circ Genom Precis Med. 2018; 11:e001937. 10.1161/CIRCGEN.117.001937 29555670 PMC5863591

[11] North BJ, Sinclair DA. The intersection between aging and cardiovascular disease. Circ Res. 2012; 110:1097–108. 10.1161/CIRCRESAHA.111.246876 22499900 PMC3366686

[12] Hamczyk MR, Nevado RM, Barettino A, Fuster V, Andrés V. Biological Versus Chronological Aging: JACC Focus Seminar. J Am Coll Cardiol. 2020; 75:919–30. 10.1016/j.jacc.2019.11.062 32130928

[13] Expert Panel on Detection, Evaluation, and Treatment of High Blood Cholesterol in Adults. Executive Summary of The Third Report of The National Cholesterol Education Program (NCEP) Expert Panel on Detection, Evaluation, And Treatment of High Blood Cholesterol In Adults (Adult Treatment Panel III). JAMA. 2001; 285:2486–97. 10.1001/jama.285.19.2486 11368702

[14] Dominguez LJ, Barbagallo M. The biology of the metabolic syndrome and aging. Curr Opin Clin Nutr Metab Care. 2016; 19:5–11. 10.1097/MCO.0000000000000243 26560521

[15] Morley JE. The metabolic syndrome and aging. J Gerontol A Biol Sci Med Sci. 2004; 59:139–42. 10.1093/gerona/59.2.m139 14999026

[16] Baylis D, Bartlett DB, Patel HP, Roberts HC. Understanding how we age: insights into inflammaging. Longev Healthspan. 2013; 2:8. 10.1186/2046-2395-2-8 24472098 PMC3922951

[17] Hernandez-Baixauli J, Quesada-Vázquez S, Mariné-Casadó R, Gil Cardoso K, Caimari A, Del Bas JM, Escoté X, Baselga-Escudero L. Detection of Early Disease Risk Factors Associated with Metabolic Syndrome: A New Era with the NMR Metabolomics Assessment. Nutrients. 2020; 12:806. 10.3390/nu12030806 32197513 PMC7146483

[18] Silva RA, Pereira TCS, Souza AR, Ribeiro PR. (1)H NMR-based metabolite profiling for biomarker identification. Clin Chim Acta. 2020; 502:269–79. 10.1016/j.cca.2019.11.015 31778675

[19] Brindle JT, Antti H, Holmes E, Tranter G, Nicholson JK, Bethell HW, Clarke S, Schofield PM, McKilligin E, Mosedale DE, Grainger DJ. Rapid and noninvasive diagnosis of the presence and severity of coronary heart disease using 1H-NMR-based metabonomics. Nat Med. 2002; 8:1439–44. 10.1038/nm1202-802 12447357

[20] Fuertes-Martín R, Correig X, Vallvé JC, Amigó N. Title: Human Serum/Plasma Glycoprotein Analysis by ^1^H-NMR, an Emerging Method of Inflammatory Assessment. J Clin Med. 2020; 9:354. 10.3390/jcm9020354 32012794 PMC7073769

[21] Ballout RA, Remaley AT. GlycA: A New Biomarker for Systemic Inflammation and Cardiovascular Disease (CVD) Risk Assessment. J Lab Precis Med. 2020; 5:17. 10.21037/jlpm.2020.03.03 32363327 PMC7194207

[22] Connelly MA, Otvos JD, Shalaurova I, Playford MP, Mehta NN. GlycA, a novel biomarker of systemic inflammation and cardiovascular disease risk. J Transl Med. 2017; 15:219. 10.1186/s12967-017-1321-6 29078787 PMC5658936

[23] Horvath S. DNA methylation age of human tissues and cell types. Genome Biol. 2013; 14:R115. 10.1186/gb-2013-14-10-r115 24138928 PMC4015143

[24] Hannum G, Guinney J, Zhao L, Zhang L, Hughes G, Sadda S, Klotzle B, Bibikova M, Fan JB, Gao Y, Deconde R, Chen M, Rajapakse I, et al. Genome-wide methylation profiles reveal quantitative views of human aging rates. Mol Cell. 2013; 49:359–67. 10.1016/j.molcel.2012.10.016 23177740 PMC3780611

[25] Lu AT, Quach A, Wilson JG, Reiner AP, Aviv A, Raj K, Hou L, Baccarelli AA, Li Y, Stewart JD, Whitsel EA, Assimes TL, Ferrucci L, Horvath S. DNA methylation GrimAge strongly predicts lifespan and healthspan. Aging (Albany NY). 2019; 11:303–27. 10.18632/aging.101684 30669119 PMC6366976

[26] Levine ME, Lu AT, Quach A, Chen BH, Assimes TL, Bandinelli S, Hou L, Baccarelli AA, Stewart JD, Li Y, Whitsel EA, Wilson JG, Reiner AP, et al. An epigenetic biomarker of aging for lifespan and healthspan. Aging (Albany NY). 2018; 10:573–91. 10.18632/aging.101414 29676998 PMC5940111

[27] Peters MJ, Joehanes R, Pilling LC, Schurmann C, Conneely KN, Powell J, Reinmaa E, Sutphin GL, Zhernakova A, Schramm K, Wilson YA, Kobes S, Tukiainen T, et al, and NABEC/UKBEC Consortium. The transcriptional landscape of age in human peripheral blood. Nat Commun. 2015; 6:8570. 10.1038/ncomms9570 26490707 PMC4639797

[28] Irvin MR, Aslibekyan S, Do A, Zhi D, Hidalgo B, Claas SA, Srinivasasainagendra V, Horvath S, Tiwari HK, Absher DM, Arnett DK. Metabolic and inflammatory biomarkers are associated with epigenetic aging acceleration estimates in the GOLDN study. Clin Epigenetics. 2018; 10:56. 10.1186/s13148-018-0481-4 29713391 PMC5907301

[29] Gao T, Wilkins JT, Zheng Y, Joyce BT, Jacobs DR Jr, Schreiner PJ, Horvath S, Greenland P, Lloyd-Jones D, Hou L. Plasma lipid profiles in early adulthood are associated with epigenetic aging in the Coronary Artery Risk Development in Young Adults (CARDIA) Study. Clin Epigenetics. 2022; 14:16. 10.1186/s13148-021-01222-2 35101102 PMC8805309

[30] Huang RC, Lillycrop KA, Beilin LJ, Godfrey KM, Anderson D, Mori TA, Rauschert S, Craig JM, Oddy WH, Ayonrinde OT, Pennell CE, Holbrook JD, Melton PE. Epigenetic Age Acceleration in Adolescence Associates With BMI, Inflammation, and Risk Score for Middle Age Cardiovascular Disease. J Clin Endocrinol Metab. 2019; 104:3012–24. 10.1210/jc.2018-02076 30785999 PMC6555851

[31] Jiang R, Hauser ER, Kwee LC, Shah SH, Regan JA, Huebner JL, Kraus VB, Kraus WE, Ward-Caviness CK. The association of accelerated epigenetic age with all-cause mortality in cardiac catheterization patients as mediated by vascular and cardiometabolic outcomes. Clin Epigenetics. 2022; 14:165. 10.1186/s13148-022-01380-x 36461124 PMC9719253

[32] Lee LJ, Shamburek R, Son H, Wallen GR, Cox R, Flynn S, Yang L, Bevans M, Wehrlen L, Ross A. Effects of a yoga-based stress reduction intervention on stress, psychological outcomes and cardiometabolic biomarkers in cancer caregivers: A randomized controlled trial. PLoS One. 2022; 17:e0277009. 10.1371/journal.pone.0277009 36355827 PMC9648784

[33] Wu L, Kong Q, Huang H, Xu S, Qu W, Zhang P, Yu Z, Luo X. Effect of PCSK9 inhibition in combination with statin therapy on intracranial atherosclerotic stenosis: A high-resolution MRI study. Front Aging Neurosci. 2023; 15:1127534. 10.3389/fnagi.2023.1127534 36967822 PMC10033935

[34] Nakajima A, Eguchi Y, Yoneda M, Imajo K, Tamaki N, Suganami H, Nojima T, Tanigawa R, Iizuka M, Iida Y, Loomba R. Randomised clinical trial: Pemafibrate, a novel selective peroxisome proliferator-activated receptor α modulator (SPPARMα), versus placebo in patients with non-alcoholic fatty liver disease. Aliment Pharmacol Ther. 2021; 54:1263–77. 10.1111/apt.16596 34528723 PMC9292296

[35] Christoffersen M, Frikke-Schmidt R, Schnohr P, Jensen GB, Nordestgaard BG, Tybjærg-Hansen A. Visible age-related signs and risk of ischemic heart disease in the general population: a prospective cohort study. Circulation. 2014; 129:990–8. 10.1161/CIRCULATIONAHA.113.001696 24334176

[36] Pal A, Rath PC. Metabolic Diseases and Aging. In: Rath P. (eds). Models, Molecules and Mechanisms in Biogerontology. Springer: Singapore. 2020. 10.1007/978-981-32-9005-1_17

[37] Joyce BT, Gao T, Zheng Y, Ma J, Hwang SJ, Liu L, Nannini D, Horvath S, Lu AT, Bai Allen N, Jacobs DR Jr, Gross M, Krefman A, et al. Epigenetic Age Acceleration Reflects Long-Term Cardiovascular Health. Circ Res. 2021; 129:770–81. 10.1161/CIRCRESAHA.121.318965 34428927 PMC8484046

[38] Pottinger TD, Khan SS, Zheng Y, Zhang W, Tindle HA, Allison M, Wells G, Shadyab AH, Nassir R, Martin LW, Manson JE, Lloyd-Jones DM, Greenland P, et al. Association of cardiovascular health and epigenetic age acceleration. Clin Epigenetics. 2021; 13:42. 10.1186/s13148-021-01028-2 33632308 PMC7905851

[39] Lind L, Ingelsson E, Sundström J, Siegbahn A, Lampa E. Methylation-based estimated biological age and cardiovascular disease. Eur J Clin Invest. 2018; 48. 10.1111/eci.12872 29231988

[40] Vona R, Gambardella L, Cittadini C, Straface E, Pietraforte D. Biomarkers of Oxidative Stress in Metabolic Syndrome and Associated Diseases. Oxid Med Cell Longev. 2019; 2019:8267234. 10.1155/2019/8267234 31191805 PMC6525823

[41] Flores-Guerrero JL, Gruppen EG, Connelly MA, Shalaurova I, Otvos JD, Garcia E, Bakker SJL, Dullaart RPF. A Newly Developed Diabetes Risk Index, Based on Lipoprotein Subfractions and Branched Chain Amino Acids, is Associated with Incident Type 2 Diabetes Mellitus in the PREVEND Cohort. J Clin Med. 2020; 9:278. 10.3390/jcm9092781 32867285 PMC7563197

[42] Sniderman AD, Thanassoulis G, Glavinovic T, Navar AM, Pencina M, Catapano A, Ference BA. Apolipoprotein B Particles and Cardiovascular Disease: A Narrative Review. JAMA Cardiol. 2019; 4:1287–95. 10.1001/jamacardio.2019.3780 31642874 PMC7369156

[43] McCrory C, Fiorito G, Hernandez B, Polidoro S, O'Halloran AM, Hever A, Ni Cheallaigh C, Lu AT, Horvath S, Vineis P, Kenny RA. GrimAge Outperforms Other Epigenetic Clocks in the Prediction of Age-Related Clinical Phenotypes and All-Cause Mortality. J Gerontol A Biol Sci Med Sci. 2021; 76:741–9. 10.1093/gerona/glaa286 33211845 PMC8087266

[44] Gruppen EG, Connelly MA, Otvos JD, Bakker SJ, Dullaart RP. A novel protein glycan biomarker and LCAT activity in metabolic syndrome. Eur J Clin Invest. 2015; 45:850–9. 10.1111/eci.12481 26081900

[45] Black S, Kushner I, Samols D. C-reactive Protein. J Biol Chem. 2004; 279:48487–90. 10.1074/jbc.R400025200 15337754

[46] Otvos JD, Shalaurova I, Wolak-Dinsmore J, Connelly MA, Mackey RH, Stein JH, Tracy RP. GlycA: A Composite Nuclear Magnetic Resonance Biomarker of Systemic Inflammation. Clin Chem. 2015; 61:714–23. 10.1373/clinchem.2014.232918 25779987

[47] Huang LH, Elvington A, Randolph GJ. The role of the lymphatic system in cholesterol transport. Front Pharmacol. 2015; 6:182. 10.3389/fphar.2015.00182 26388772 PMC4557107

[48] Cochran BJ, Ong KL, Manandhar B, Rye KA. APOA1: a Protein with Multiple Therapeutic Functions. Curr Atheroscler Rep. 2021; 23:11. 10.1007/s11883-021-00906-7 33591433

[49] Zamanian-Daryoush M, Lindner D, Tallant TC, Wang Z, Buffa J, Klipfell E, Parker Y, Hatala D, Parsons-Wingerter P, Rayman P, Yusufishaq MSS, Fisher EA, Smith JD, et al. The cardioprotective protein apolipoprotein A1 promotes potent anti-tumorigenic effects. J Biol Chem. 2013; 288:21237–52. 10.1074/jbc.M113.468967 23720750 PMC3774392

[50] Shah GN, Wong NC, Mooradian AD. Age-related changes in apolipoprotein A-I expression. Biochim Biophys Acta. 1995; 1259:277–82. 10.1016/0005-2760(95)00174-3 8541335

[51] Lu J, Huang Y, Wang Y, Li Y, Zhang Y, Wu J, Zhao F, Meng S, Yu X, Ma Q, Song M, Chang N, Bittles AH, Wang W. Profiling plasma peptides for the identification of potential ageing biomarkers in Chinese Han adults. PLoS One. 2012; 7:e39726. 10.1371/journal.pone.0039726 22802942 PMC3389038

[52] Kraus WE, Granger CB, Sketch MH Jr, Donahue MP, Ginsburg GS, Hauser ER, Haynes C, Newby LK, Hurdle M, Dowdy ZE, Shah SH. A Guide for a Cardiovascular Genomics Biorepository: the CATHGEN Experience. J Cardiovasc Transl Res. 2015; 8:449–57. 10.1007/s12265-015-9648-y 26271459 PMC4651812

[53] Houseman EA, Accomando WP, Koestler DC, Christensen BC, Marsit CJ, Nelson HH, Wiencke JK, Kelsey KT. DNA methylation arrays as surrogate measures of cell mixture distribution. BMC Bioinformatics. 2012; 13:86. 10.1186/1471-2105-13-86 22568884 PMC3532182

[54] Jeyarajah EJ, Cromwell WC, Otvos JD. Lipoprotein particle analysis by nuclear magnetic resonance spectroscopy. Clin Lab Med. 2006; 26:847–70. 10.1016/j.cll.2006.07.006 17110242

[55] McGarrah RW, Kelly JP, Craig DM, Haynes C, Jessee RC, Huffman KM, Kraus WE, Shah SH. A Novel Protein Glycan-Derived Inflammation Biomarker Independently Predicts Cardiovascular Disease and Modifies the Association of HDL Subclasses with Mortality. Clin Chem. 2017; 63:288–96. 10.1373/clinchem.2016.261636 27811210 PMC5429588

[56] Whitfield AJ, Barrett PH, van Bockxmeer FM, Burnett JR. Lipid disorders and mutations in the APOB gene. Clin Chem. 2004; 50:1725–32. 10.1373/clinchem.2004.038026 15308601

[57] Sattar N, Williams K, Sniderman AD, D'Agostino R Jr, Haffner SM. Comparison of the associations of apolipoprotein B and non-high-density lipoprotein cholesterol with other cardiovascular risk factors in patients with the metabolic syndrome in the Insulin Resistance Atherosclerosis Study. Circulation. 2004; 110:2687–93. 10.1161/01.CIR.0000145660.60487.94 15492304

[58] Marston NA, Giugliano RP, Melloni GEM, Park JG, Morrill V, Blazing MA, Ference B, Stein E, Stroes ES, Braunwald E, Ellinor PT, Lubitz SA, Ruff CT, Sabatine MS. Association of Apolipoprotein B-Containing Lipoproteins and Risk of Myocardial Infarction in Individuals With and Without Atherosclerosis: Distinguishing Between Particle Concentration, Type, and Content. JAMA Cardiol. 2022; 7:250–6. 10.1001/jamacardio.2021.5083 34773460 PMC8590731

[59] van der Vorst EPC. High-Density Lipoproteins and Apolipoprotein A1. Subcell Biochem. 2020; 94:399–420. 10.1007/978-3-030-41769-7_16 32189309

[60] Gao L, Zhang Y, Wang X, Dong H. Association of apolipoproteins A1 and B with type 2 diabetes and fasting blood glucose: a cross-sectional study. BMC Endocr Disord. 2021; 21:59. 10.1186/s12902-021-00726-5 33794863 PMC8017773

[61] Dong H, Yang X, Zhang Y, Hu P, Liu Y, Liang S. Associations of serum apolipoprotein A1, B levels and their ratio with blood pressure in Chinese adults with coronary artery disease. Blood Press Monit. 2021; 26:401–6. 10.1097/MBP.0000000000000549 34074807

[62] Jing F, Mao Y, Guo J, Zhang Z, Li Y, Ye Z, Ding Y, Wang J, Jin M, Chen K. The value of Apolipoprotein B/Apolipoprotein A1 ratio for metabolic syndrome diagnosis in a Chinese population: a cross-sectional study. Lipids Health Dis. 2014; 13:81. 10.1186/1476-511X-13-81 24886173 PMC4041140

[63] Lu M, Lu Q, Zhang Y, Tian G. ApoB/apoA1 is an effective predictor of coronary heart disease risk in overweight and obesity. J Biomed Res. 2011; 25:266–73. 10.1016/S1674-8301(11)60036-5 23554700 PMC3597070

[64] Shalaurova I, Connelly MA, Garvey WT, Otvos JD. Lipoprotein insulin resistance index: a lipoprotein particle-derived measure of insulin resistance. Metab Syndr Relat Disord. 2014; 12:422–9. 10.1089/met.2014.0050 24959989 PMC4175429

[65] Dugani SB, Akinkuolie AO, Paynter N, Glynn RJ, Ridker PM, Mora S. Association of Lipoproteins, Insulin Resistance, and Rosuvastatin With Incident Type 2 Diabetes Mellitus : Secondary Analysis of a Randomized Clinical Trial. JAMA Cardiol. 2016; 1:136–45. 10.1001/jamacardio.2016.0096 27347563 PMC4918085

[66] Hu W, Yang P, Fu Z, Wang Y, Zhou Y, Ye Z, Gong Y, Huang A, Sun L, Zhao Y, Yang T, Li Z, Jiang XC, et al. High L-Valine Concentrations Associate with Increased Oxidative Stress and Newly-Diagnosed Type 2 Diabetes Mellitus: A Cross-Sectional Study. Diabetes Metab Syndr Obes. 2022; 15:499–509. 10.2147/DMSO.S336736 35221701 PMC8865866

[67] Liao X, Liu B, Qu H, Zhang L, Lu Y, Xu Y, Lyu Z, Zheng H. A High Level of Circulating Valine Is a Biomarker for Type 2 Diabetes and Associated with the Hypoglycemic Effect of Sitagliptin. Mediators Inflamm. 2019; 2019:8247019. 10.1155/2019/8247019 31827381 PMC6885205

[68] Ward-Caviness CK, Russell AG, Weaver AM, Slawsky E, Dhingra R, Kwee LC, Jiang R, Neas LM, Diaz-Sanchez D, Devlin RB, Cascio WE, Olden K, Hauser ER, et al. Accelerated epigenetic age as a biomarker of cardiovascular sensitivity to traffic-related air pollution. Aging (Albany NY). 2020; 12:24141–55. 10.18632/aging.202341 33289704 PMC7762491

[69] McGuinn LA, Schneider A, McGarrah RW, Ward-Caviness C, Neas LM, Di Q, Schwartz J, Hauser ER, Kraus WE, Cascio WE, Diaz-Sanchez D, Devlin RB. Association of long-term PM_2.5_ exposure with traditional and novel lipid measures related to cardiovascular disease risk. Environ Int. 2019; 122:193–200. 10.1016/j.envint.2018.11.001 30446244 PMC6467069

[70] Zhang S, Breitner S, Cascio WE, Devlin RB, Neas LM, Ward-Caviness C, Diaz-Sanchez D, Kraus WE, Hauser ER, Schwartz J, Peters A, Schneider A. Association between short-term exposure to ambient fine particulate matter and myocardial injury in the CATHGEN cohort. Environ Pollut. 2021; 275:116663. 10.1016/j.envpol.2021.116663 33581627

