# Supplementary material for: Associations among NMR-measured inflammatory and metabolic biomarkers and accelerated aging in cardiac catheterization patients
Source: Aging (Albany NY). 2024 Apr 23;16(8):6652–72. doi: 10.18632/aging.205758 (PMC11087135; doi:10.18632/aging.205758)
Supplement: Supplementary Figure 1 [file aging-16-205758-s001.pdf]

## SUPPLEMENTARY FIGURE

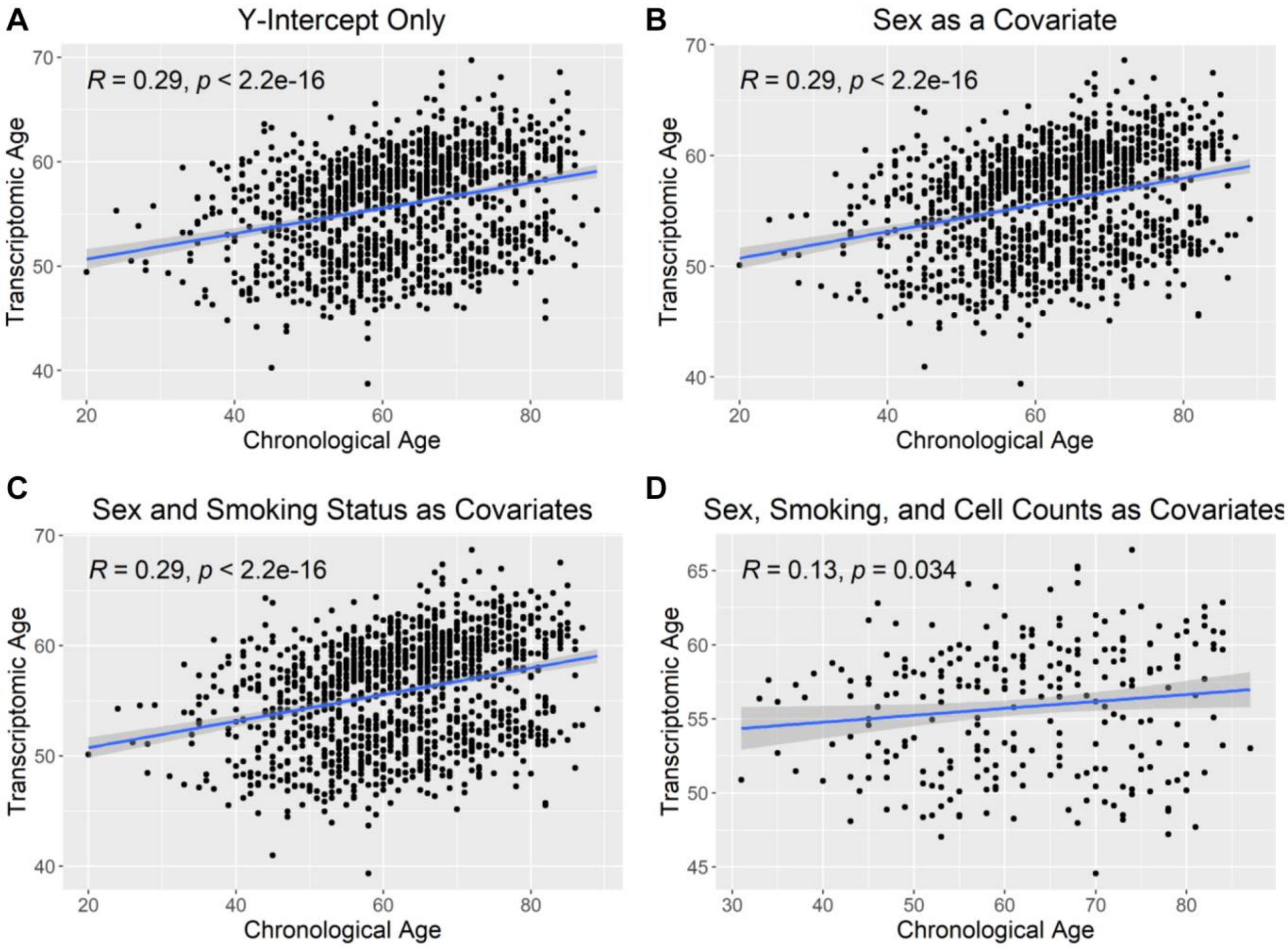


**Supplementary Figure 1. Correlation between chronological age and transcriptomic age when transcriptomic age was calculated using different covariates.** The scatterplots show the correlation between chronological age and transcriptomic age. (**A**–**D**) show these correlations using residuals from models for estimating transcriptomic age where the models differ in the included covariates. In (**A**) no additional covariates were included beyond the intercept (which was included in all models). In (**B**) the model was adjusted for sex. In (**C**) the model was adjusted for sex and smoking status. Finally, in (**D**) the model was adjusted for sex, smoking status, and cell counts. R = Pearson correlation coefficient.
