# Supplementary material for: Associations among NMR-measured inflammatory and metabolic biomarkers and accelerated aging in cardiac catheterization patients
Source: Aging (Albany NY). 2024 Apr 23;16(8):6652–72. doi: 10.18632/aging.205758 (PMC11087135; doi:10.18632/aging.205758)
Supplement: Supplementary Tables [file aging-16-205758-s002.pdf]

SUPPLEMENTARY TABLES

Supplementary Table 1. Associations between accelerated ages of epigenetic and transcriptomic biomarkers and nuclear magnetic resonance biomarkers.

Biomarker	Aging predictor	Coefficient (95% CI)	*P*-value
GlycA	GrimAge	5.71 (4.36, 7.05)	$7.94 \times 10^{-16*}$
ApoB	GrimAge	0.10 (−0.35, 0.55)	0.65
ApoA-1	GrimAge	−1.03 (−1.42, −0.63)	$5.50 \times 10^{-7*}$
Glu	GrimAge	−0.12 (−0.82, 0.59)	0.74
ApoB: A-1	GrimAge	0.02 (−0.01, 0.06)	0.20
GlycA	Hannum Age	1.81 (0.65, 2.98)	2.30×10^{-3}
ApoB	Hannum Age	−0.06 (−0.42, 0.31)	0.76
ApoA-1	Hannum Age	−0.41 (−0.74, −0.08)	0.01
Glu	Hannum Age	0.37 (−0.20, 0.94)	0.21
ApoB: A-1	Hannum Age	0.01 (−0.02, 0.04)	0.47
GlycA	Horvath Age	0.81 (−0.45, 2.07)	0.21
ApoB	Horvath Age	−0.19 (−0.58, 0.21)	0.36
ApoA-1	Horvath Age	−0.29 (−0.64, 0.07)	0.12
Glu	Horvath Age	0.28 (−0.34, 0.89)	0.37
ApoB: A-1	Horvath Age	-5.22×10^{-3} (−0.03, 0.02)	0.73
GlycA	Phenotypic Age	2.88 (1.91, 3.87)	$1.21 \times 10^{-8*}$
ApoB	Phenotypic Age	0.07 (−0.25, 0.39)	0.67
ApoA-1	Phenotypic Age	−0.55 (−0.83, −0.26)	$1.58 \times 10^{-4*}$
Glu	Phenotypic Age	0.12 (−0.38, 0.61)	0.64
ApoB: A-1	Phenotypic Age	8.96×10^{-3} (−0.01, 0.03)	0.46
GlycA	Transcriptomic Age	0.34 (−0.85, 1.53)	0.57
ApoB	Transcriptomic Age	0.05 (−0.26, 0.37)	0.74
ApoA-1	Transcriptomic Age	0.34 (0.04, 0.63)	0.03
Glu	Transcriptomic Age	0.23 (−0.36, 0.82)	0.45
ApoB: A-1	Transcriptomic Age	-1.27×10^{-3} (-5.29×10^{-3}, 2.75×10^{-3})	0.54

Abbreviation: CI: confidence interval; *indicates Bonferroni significant *P*-value ($P < 1.43 \times 10^{-3}$).

Supplementary Table 2. Associations between nuclear magnetic resonance biomarkers and multiple adjusted accelerated ages of epigenetic and transcriptomic aging biomarkers.

Biomarker	Aging predictor adjustment	Aging predictor	Coefficient (95% CI)	*P*-value
GlycA	Gr + Ha + Ho + Ph + TA	GrimAge	5.03 (3.01, 7.05)	$1.85 \times 10^{6*}$
ApoB	Gr + Ha + Ho + Ph + TA	GrimAge	0.45 (−0.22, 1.11)	0.19
ApoA-1	Gr + Ha + Ho + Ph + TA	GrimAge	−1.13 (−1.71, −0.55)	$1.83 \times 10^{-4*}$
Glu	Gr + Ha + Ho + Ph + TA	GrimAge	−0.09 (−1.16, 0.99)	0.87
ApoB: A-1	Gr + Ha + Ho + Ph + TA	GrimAge	1.04×10^{-2} (2.9×10^{-3}, 0.02)	6.84×10^{-3}
GlycA	Gr + Ha + Ho + Ph + TA	Hannum Age	1.17 (−0.77, 3.12)	0.24
ApoB	Gr + Ha + Ho + Ph + TA	Hannum Age	0.24 (−0.40, 0.88)	0.46
ApoA-1	Gr + Ha + Ho + Ph + TA	Hannum Age	−0.60 (−1.16, −0.04)	0.04
Glu	Gr + Ha + Ho + Ph + TA	Hannum Age	0.21 (−0.82, 1.25)	0.68
ApoB: A-1	Gr + Ha + Ho + Ph + TA	Hannum Age	6.27×10^{-3} (-9.74×10^{-4}, 0.01)	0.09
GlycA	Gr + Ha + Ho + Ph + TA	Horvath Age	−2.94 (−4.86, −1.02)	2.89×10^{-3}

ApoB	Gr + Ha + Ho + Ph + TA	Horvath Age	−0.80 (−1.43, −0.16)	0.01
ApoA-1	Gr + Ha + Ho + Ph + TA	Horvath Age	−0.12 (−0.67, 0.44)	0.67
Glu	Gr + Ha + Ho + Ph + TA	Horvath Age	0.13 (−0.89, 1.15)	0.80
ApoB: A-1	Gr + Ha + Ho + Ph + TA	Horvath Age	-6.89×10^{-3} (-0.01, 2.66×10^{-4})	0.06
GlycA	Gr + Ha + Ho + Ph + TA	Phenotypic Age*	2.27 (0.47, 4.06)	0.01
ApoB	Gr + Ha + Ho + Ph + TA	Phenotypic Age	0.18 (−0.41, 0.77)	0.55
ApoA-1	Gr + Ha + Ho + Ph + TA	Phenotypic Age	0.28 (−0.24, 0.80)	0.29
Glu	Gr + Ha + Ho + Ph + TA	Phenotypic Age	0.33 (−0.62, 1.29)	0.49
ApoB: A-1	Gr + Ha + Ho + Ph + TA	Phenotypic Age	-2.30×10^{-4} (-6.93×10^{-3}, 6.47×10^{-3})	0.95
GlycA	Gr + Ha + Ho + Ph + TA	Transcriptomic Age	−0.85 (−2.90, 1.13)	0.39
ApoB	Gr + Ha + Ho + Ph + TA	Transcriptomic Age	0.08 (−0.58, 0.75)	0.80
ApoA-1	Gr + Ha + Ho + Ph + TA	Transcriptomic Age	0.56 (−0.02, 1.15)	0.06
Glu	Gr + Ha + Ho + Ph + TA	Transcriptomic Age	−0.13 (−1.20, 0.94)	0.81
ApoB: A-1	Gr + Ha + Ho + Ph + TA	Transcriptomic Age	-3.86×10^{-3} (-0.01, 3.66×10^{-3})	0.31

Abbreviations: Gr: Grim Age; Ha: Hannum Age; Ho: Horvath Age; Ph: Phenotypic Age; TA: Transcriptomic Age; CI: confidence interval. *indicates Bonferroni significant *P*-value ($P < 1.43 \times 10^{-3}$).

Supplementary Table 3. Associations between nuclear magnetic resonance biomarkers and multiple adjusted accelerated ages of epigenetic aging biomarkers.

Biomarker	Aging predictor adjustment	Aging predictor	Coefficient (95% CI)	*P*-value
GlycA	Gr + Ha + Ho + Ph	GrimAge	5.03 (3.01, 7.05)	1.83×10^{-6}*
ApoB	Gr + Ha + Ho + Ph	GrimAge	0.45 (−0.22, 1.11)	0.19
ApoA-1	Gr + Ha + Ho + Ph	GrimAge	−1.13 (−1.72, −0.54)	2.02×10^{-4}*
Glu	Gr + Ha + Ho + Ph	GrimAge	−0.08 (−1.16, 0.98)	0.87
ApoB: A-1	Gr + Ha + Ho + Ph	GrimAge	0.01 (2.89×10^{-3}, 1.79×10^{-2})	6.88×10^{-3}
GlycA	Gr + Ha + Ho + Ph	Hannum Age	1.07 (−0.86, 2.99)	0.28
ApoB	Gr + Ha + Ho + Ph	Hannum Age	0.25 (−0.38, 0.89)	0.43
ApoA-1	Gr + Ha + Ho + Ph	Hannum Age	−0.53 (−1.09, 0.03)	0.06
Glu	Gr + Ha + Ho + Ph	Hannum Age	0.20 (−0.82, 1.22)	0.70
ApoB: A-1	Gr + Ha + Ho + Ph	Hannum Age	5.80×10^{-3} (-1.39×10^{-3}, 0.01)	0.11
GlycA	Gr + Ha + Ho + Ph	Horvath Age	−2.90 (−4.81, −0.98)	3.23×10^{-3}
ApoB	Gr + Ha + Ho + Ph	Horvath Age	−0.80 (−1.43, −0.17)	0.01
ApoA-1	Gr + Ha + Ho + Ph	Horvath Age	−0.14 (−0.70, 0.42)	0.61
Glu	Gr + Ha + Ho + Ph	Horvath Age	0.14 (−0.88, 1.15)	0.79
ApoB: A-1	Gr + Ha + Ho + Ph	Horvath Age	-6.72×10^{-3} (-0.01, 4.28×10^{-4})	0.07
GlycA	Gr + Ha + Ho + Ph	Phenotypic Age	2.26 (0.46, 4.05)	0.01
ApoB	Gr + Ha + Ho + Ph	Phenotypic Age	0.18 (−0.41, 0.77)	0.55
ApoA-1	Gr + Ha + Ho + Ph	Phenotypic Age	0.29 (−0.24, 0.81)	0.28
Glu	Gr + Ha + Ho + Ph	Phenotypic Age	0.33 (−0.62, 1.28)	0.50
ApoB: A-1	Gr + Ha + Ho + Ph	Phenotypic Age	$-2.63 x 10^{-4}$ (-7.0×10^{-3}, 6.43×10^{-3})	0.94

Abbreviations: Gr: Grim Age; Ho: Horvath Age; Ha: Hannum Age; Ph: Phenotypic Age; CI: confidence interval. *indicates Bonferroni significant *P*-value ($P < 1.43 \times 10^{-3}$).

Supplementary Table 4. Associations between accelerated ages of individual epigenetic and transcriptomic aging predictors and nuclear magnetic resonance multi markers.

Biomarker	Aging predictor adjustment	Aging predictor	Coefficient (95% CI)	*P*-value
LP-IR	Grim Age	GrimAge	0.18 (−0.20, 0.56)	0.35
DRI	Grim Age	GrimAge	−0.29 (−0.57, -2.03×10^{-3})	0.05
LP-IR	Hannum Age	Hannum Age	0.15 (−0.16, 0.46)	0.34
DRI	Hannum Age	Hannum Age	−0.04 (−0.28, 0.19)	0.72
LP-IR	Horvath Age	Horvath Age	0.32 (-9.36×10^{-3}, 0.66)	0.06
DRI	Horvath Age	Horvath Age	0.06 (−0.20, 0.31)	0.67
LP-IR	Phenotypic Age	Phenotypic Age	0.18 (−0.08, 0.45)	0.18
DRI	Phenotypic Age	Phenotypic Age	−0.09 (−0.29, 0.15)	0.40
LP-IR	Transcriptomic Age	Transcriptomic Age	−0.13 (−0.44, 0.18)	0.40
DRI	Transcriptomic Age	Transcriptomic Age	−0.02 (−0.26, 0.23)	0.88

Abbreviation: CI: confidence interval.

Supplementary Table 5. Associations between nuclear magnetic resonance multi markers and multiple adjusted accelerated ages of epigenetic and transcriptomic aging biomarkers.

Biomarker	Aging predictor adjustment	Aging predictor	Coefficient (95% CI)	*P*-value
LP-IR	Gr + Ha + Ho + Ph + TA	GrimAge	0.32 (−0.28, 0.93)	0.29
DRI	Gr + Ha + Ho + Ph + TA	GrimAge	−0.22 (−0.68, 0.24)	0.35
LP-IR	Gr + Ha + Ho + Ph + TA	Hannum Age	0.16 (−0.42, 0.74)	0.59
DRI	Gr + Ha + Ho + Ph + TA	Hannum Age	−0.01 (−0.46, 0.43)	0.95
LP-IR	Gr + Ha + Ho + Ph + TA	Horvath Age	0.13 (−0.44, 0.71)	0.64
DRI	Gr + Ha + Ho + Ph + TA	Horvath Age	0.04 (−0.40, 0.48)	0.87
LP-IR	Gr + Ha + Ho + Ph + TA	Phenotypic Age	−0.12 (−0.66, 0.42)	0.66
DRI	Gr + Ha + Ho + Ph + TA	Phenotypic Age	−0.10 (−0.51, 0.31)	0.64
LP-IR	Gr + Ha + Ho + Ph + TA	Transcriptomic Age	−0.11 (−0.72, 0.49)	0.71
DRI	Gr + Ha + Ho + Ph + TA	Transcriptomic Age	−0.08 (−0.55, 0.38)	0.72

Abbreviations: Gr: Grim Age; Ha: Hannum Age; Ho: Horvath Age; Ph: Phenotypic Age; TA: Transcriptomic Age; CI: confidence interval.

Supplementary Table 6. Associations between nuclear magnetic resonance multi markers and multiple adjusted accelerated ages of epigenetic aging predictors.

Biomarker	Aging predictor adjustment	Aging predictor	Coefficient (95% CI)	*P*-value
LP-IR	Gr + Ha + Ho + Ph	GrimAge	0.32 (−0.35, 0.93)	0.29
DRI	Gr + Ha + Ho + Ph	GrimAge	−0.22 (−0.68, 0.24)	0.35
LP-IR	Gr + Ha + Ho + Ph	Hannum Age	0.15 (−0.43, 0.72)	0.62
DRI	Gr + Ha + Ho + Ph	Hannum Age	−0.03 (−0.46, 0.41)	0.91
LP-IR	Gr + Ha + Ho + Ph	Horvath Age	0.14 (−0.43, 0.71)	0.63
DRI	Gr + Ha + Ho + Ph	Horvath Age	−0.04 (−0.40, 0.48)	0.85
LP-IR	Gr + Ha + Ho + Ph	Phenotypic Age	−0.12 (−0.65, 0.42)	0.66
DRI	Gr + Ha + Ho + Ph	Phenotypic Age	−0.10 (−0.51, 0.31)	0.64

Abbreviations: Gr: Grim Age; Ha: Hannum Age; Ho: Horvath Age; Ph: Phenotypic Age; CI: confidence interval.

Supplementary Table 7. Associations between LDL and HDL and accelerated ages of individual epigenetic and transcriptomic aging biomarkers.

Biomarker	Aging predictor	Coefficient (95% CI)	*P*-value
HDL	GrimAge	−0.33 (−0.68, 0.03)	0.07
LDL	GrimAge	−0.22 (−1.34, 0.90)	0.70
HDL	Hannum Age	−0.32 (−0.65, 6.28×10^{-3})	0.05
LDL	Hannum Age	−1.11 (−2.16, −0.07)	0.04
HDL	Horvath Age	−0.29 (−0.62, 0.04)	0.08
LDL	Horvath Age	−1.13 (−2.15, −0.10)	0.03
HDL	Phenotypic Age	−0.30 (−0.56, −0.04)	0.03
LDL	Phenotypic Age	−0.74 (−1.56, 0.09)	0.08
HDL	Transcriptomic Age	−0.18 (−0.12, 0.48)	0.25
LDL	Transcriptomic Age	0.24 (−0.61, 1.09)	0.58

Abbreviation: CI: confidence interval.
